# 

**Published:** 1885-07

**Authors:** 


### BOOK NOTICES.

MANIPULATION OF THE MICROSCOPE.—Edward Bausch. We are in receipt of a little book with the above title. The treatment of the manipulation of the microscope could be in no more competent hands than Mr. Bausch, and we deem it only necessary to inform microscopists that such a work is in existence to insure a rapid exhaustion of the present edition and a call for more.

THE TECHNOLOGY OF BACTERIA INVESTIGATION.—Charles S. Dolley, M. D. Boston, S. E. Cassino & Co., This work of special interest at the present time indeed at all times, has been written by its able author with great care and contains explicit directions for the study of Bacteria, their culture, staining, mounting &c, according to the methods in use by the most eminent investigators. It is published at two dollars and may be had of all booksellers, we may have occasion to refer at a future time more particularly to its contents.

### ⋉Dr. Up de Graff's Eye and Ear Institute,⋊

ELMIRA, N. Y.

DEAR SIR:

On account of greater professional responsibilities of late, I am obliged to discontinue the publication of "The Bistoury."

I regret very much that I cannot spare time for its continuance.

Those who have paid their subscription in advance have had their money refunded.

Very truly,

Thad S. Up de Graff, M. D.

